# Characterization of inflorescence-predominant *chitinase* gene in *Metroxylon sagu* via differential display

**DOI:** 10.1007/s13205-011-0004-x

**Published:** 2011-05-03

**Authors:** Hairul Azman Roslan, Syahrul Bariyah Anji

**Affiliations:** Department of Molecular Biology, Faculty of Resource Science and Technology, Universiti Malaysia Sarawak, 94300 Kota Samarahan, Sarawak Malaysia

**Keywords:** Chitinase, Metroxylon sagu, RACE-PCR, Differential display, Sago palm, Inflorescence-dominant

## Abstract

Chitinase is an enzyme that catalyzes the degradation of chitin, commonly induced upon the attack of pathogens and other stresses. A cDNA (*MsChi*1) was isolated from *Metroxylon sagu* and expressed predominantly in the inflorescence tissue of *M. sagu,* suggesting its role in developmental processes. The *chitinase* cDNA was detected and isolated via differential display and rapid amplification of cDNA ends (RACE). Primers specific to *M. saguchitinase* were used as probes to amplify the 3′-end and 5′-end regions of *chitinase* cDNA. Transcript analysis showed that *chitinase* is expressed in inflorescence and meristem tissues but was not detected in the leaf tissue. Sequence analysis of amplified cDNA fragments of 3′-end and 5′-end regions indicated that the *chitinase* cDNA was successfully amplified. The *M. saguchitinase* cDNA isolated was approximately 1,143 bp long and corresponds to 312 predicted amino acids. Alignments of nucleotide and amino acid have grouped this *chitinase* to family 19 class I *chitinase*.

## Introduction

*Metroxylon sagu* or locally known as sago palm, belongs to the *Lepidocaryoid* subfamily of *Arecaceae* (*Palmae*). It is a once-flowering (hapaxanthic), tillering or suckering perennial that thrives in swamp areas. The trunk of the *M. sagu* is used to obtain starch for human consumption (Flach 1984) and it is an important plant contributing to the economy of Sarawak. The advantages of *M. sagu* cultivation are that it requires no fertilization, has few natural pests or diseases and can be grown in swamp areas where it is impossible for other crops to thrive (Abd-Aziz 2002).

Chitinases are proteins that catalyze the hydrolysis of β-1,4-linkages of *N*-acetyl-d-glucosamine polymer of chitin; a major component of the exoskeleton of insects, crustacean shells and cell wall of many fungi (Bishop et al. 2000; El-Sayed et al. 2000; Passarinho and de Vries 2002). Chitinases are present in many higher plant species, although higher plants themselves do not contain chitin, chitosan or chitin-like substrate (Boller et al. 1983; Hirano et al. 1988). Chitinases are often described as pathogenesis-related proteins because they are constitutively expressed at low levels and increase dramatically in response to fungal, bacteria or viral infections (Graham and Sticklen 1994; van Loon 1999). Chitinases also play a role in plant defense mechanism by damaging chitin structures of parasites (Bishop et al. 2000; Odjakova and Hadjiivanova 2001). Apart from that, *chitinase* can also be induced by stress or elicitors such as wounding, salicylic acid and ethylene (Graham and Sticklen 1994; Leon et al. 2001).

Plant *chitinases* are classified in to classes I–V depending on their sequences and primary structures (Collinge et al. 1993; Neuhaus et al. 1996). Several studies have revealed that some *chitinase* are expressed at higher levels in healthy floral and flower-predominant organs such as potato (Wemmer et al. 1994) and tomato (Harikrishna et al. 1996). The expression of *chitinase* in flowers have also been detected in *Arabidopsis thaliana* (Samac et al. 1990; Passarinho et al. 2001), petunia (Leung 1992), parsley (Ponath et al. 2000), rice (Takakura et al. 2000) and tobacco (Lotan et al. 1989; Trudel and Asselin 1989; Neale et al. 1990). *Chitinase* expressions were also found in other tissues such as the roots of *Arabidopsis thaliana* (Samac and Shah 1991), rice (Lamb et al. 1991) and tobacco (Memelink et al. 1990; Neale et al. 1990); as well as in embryogenic cultures of carrot (van Hengel et al. 1998) and spruce (Egertsdotter 1996; Dong and Dunstan 1997). In other plants such as barley (Leah et al. 1994), carrot (van Hengel et al. 1998), pea (Petruzzelli et al. 1999) and soybean (Yeboah et al. 1998) the *chitinase* gene was detected in the seeds. Expression of *chitinase* due to infection by pathogen has also been shown in several plants such as pineapples (Taira et al. 2005) and grapes (Vasanthaiah et al. 2008).

Here we report the differential expression of *chitinase* in the leaf, meristem and inflorescence of *M. sagu*, the isolation and characterization of a near complete *chitinase* cDNA from inflorescence tissue.

## Materials and methods

### Plant materials

**Fig. 1:**
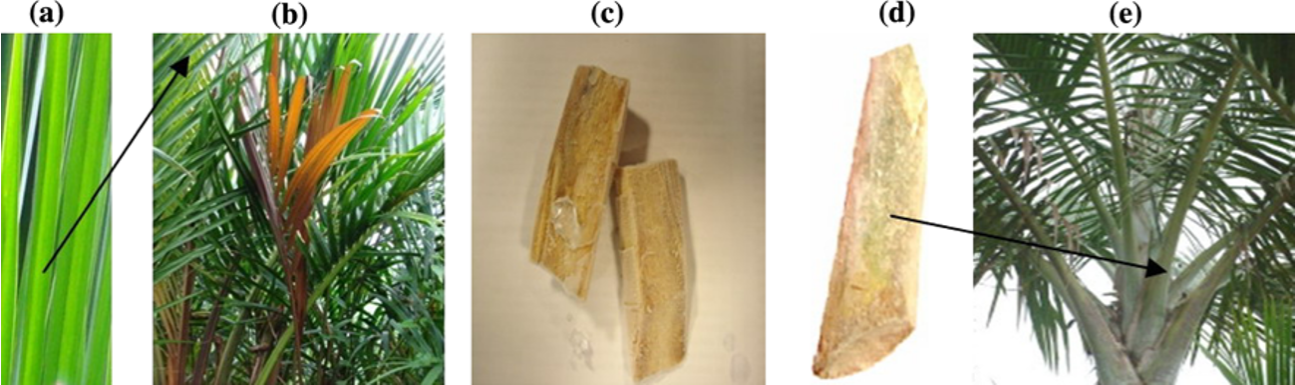
The leaves, meristem and inflorescence samples of *M. sagu*. **a** The leaf samples. **b** The vegetative growth of the palm. **c** The meristem samples. **d** The inflorescence sample. **e** The inflorescence developing palm (photo copyright CRAUN Research Sdn. Bhd, taken from Tie 2004)

Three tissue types were selected in this study: leaf, meristem and inflorescence. The leaves of *M. sagu* (Fig. 1a, b) were collected from the UNIMAS plant house. The meristem and inflorescence (Fig. 1c–e) were collected from Sri Aman areas. All the samples were stored at −80 °C,

### Ribonucleic acids (RNA) isolation and purification

Isolation of total RNA was carried out using the method described by Gasic et al. (2004), with modifications for small scale extraction. The quality and quantity of the isolated RNA were verified by agarose gel electrophoresis and spectrophotometry (Ultrospec^®^ 1100 pro, Amersham Pharmacia Biotech). The total RNA samples were treated with RQ1 RNase-Free DNase (Promega), to ensure the samples were completely free of genomic DNA contaminants (Tan and Roslan 2008).

### First-strand cDNA synthesis

First-strand cDNA was synthesized using RevertAid^TM^ Moloney Murine Leukemia Virus Reverse Transcriptase (M-MuLV RT) (Fermentas) from purified total RNA of leaf, meristem and inflorescence according to the method described by Tan and Roslan (2008). The oligo(dt)_15_ACP was used as a cDNA synthesis primer.

### Differential display reverse transcription PCR

Differential display reverse transcription PCR (DDRT-PCR) was undertaken according to the method described by Kim et al. (2004) with modifications in the PCR steps. Two annealing control primers (ACP2 and ACP3) were used to screen for differentially expressed genes in the selected tissues. PCR was performed using two combinations of ACP primers; oligo(dt)_15_ACP/AP2 and oligo(dt)_15_ACP/AP3. PCR was carried out in a final volume of 25 μl. The reaction mixture included: 2.5 μl of 10× PCR buffer, 0.25 μl of *Taq* DNA polymerase (5 U/μl) (Fermentas), 1.0 μl of each forward and reverse primers (10 μM), 1.5 μl of 25 mM MgCl_2,_ 0.5 μl of 10 mM dNTPs, sterile distilled water and 3.0 μl of 10× diluted RT product. PCR was performed using a Mastercycler Personal (Eppendorf) with thermal cycling conditions of one cycle of 94 °C for 4 min followed by five cycles at 94 °C for 1 min, 36 °C for 1 min, and 72 °C for 2 min. A further 35 cycles was undertaken at 94 °C for 1 min, 65 °C for 1 min, 72 °C for 2 min, and a 5-min final extension at 72 °C. The amplified PCR products were separated in 2.5% agarose gel stained with ethidium bromide.

### Isolation of the 3′-end of *chitinase* cDNA

The 3′ and 5′RACE were conducted according to the method described by Frohman et al. (1988). The strategy employed to isolate the *chitinase* cDNA is given in the diagram below.



The first strand cDNA was generated using oligo(dt)_17_ primer (5′-GACTCGAGTCGACATCGATTTTTTTTTTTTTTTTT-3′). A combination of emChi-f (5′-GGTGTCATCACCAACATCATCAA-3′) and oligo(dt)_17_ was used to amplify the 3′-end of the *chitinase* cDNA in meristem, inflorescence and leaf tissues of *M. sagu*. PCR amplification was carried out in a final volume of 25 μl. The reaction mixture includes: 2.5 μl of 10× PCR buffer, 0.25 μl of *Taq* DNA polymerase (5 U/μl), 0.25 μl of each primer (10 μM), 1.0 μl of 25 mM MgCl_2,_ 0.5 μl of 10 mM dNTPs, sterile water and 3.0 μl of diluted template. Thermal cycling condition was one cycle of 94 °C for 4 min; 35 cycles of 94 °C for 30 s, 65 °C for 30 s, 72 °C for 1 min; and a final extension at 72 °C for 7 min.

### Isolation of the 5′-end of *chitinase* cDNA

The first strand of cDNA from purified inflorescence total RNA was generated using gene specific primer, Chi-sp1 (5′-GCCTCTGGTTGTAGCAGTCCA-3′). The cDNA was purified and a terminal deoxynucleotidyl transferase (Fermentas) was used to tail the 3′-end of the cDNA with dATP prior to PCR amplification using Chi-sp2 (5′-GCCCTCCATTGATGATGTTG-3′) and oligo(dt)_17_ primer combination. Amplification was carried out in a final volume of 25 μl. The reaction mixture includes: 2.5 μl of 10× PCR buffer, 0.25 μl of *Taq* DNA polymerase (5 U/μl), 1.0 μl of each primer (10 μM), 1.5 μl of 25 mM MgCl_2,_ 0.5 μl of 10 mM dNTPs, sterile water and 1.0 μl of diluted template. Thermal cycling condition was one cycle of 94 °C for 4 min; 30 cycles of 94 °C for 30 s, 55 °C for 45 s, 72 °C for 1.5 min; and a final extension at 72 °C for 7 min.

### Cloning of PCR products

The purified PCR products were cloned into the pGEM-T Vector (Promega). The positive clones were screened via PCR using universal primer set T7 (5′-TAATACGACTCACTATAGGG-3′) and SP6 (5′-TATTTAGGTGACACTATAG-3′). Clones corresponding to the expected size were selected for plasmid extraction using the GeneJET™ Plasmid Miniprep Kit (Fermentas) and sequenced.

### DNA sequencing and bioinformatics analysis

Direct sequencing on plasmid DNA was performed by 1^st^ BASE Laboratories Sdn Bhd (Malaysia) using ABI PRISM^®^ 377 DNA sequencer. The sequences were analyzed using ChromasPro version 1.34 software. Alignment of nucleotide sequences was conducted using European Bioinformatics Institute’s (EBI) Clustal W multiple alignment software and similarity sequence searches were carried out using programs at the National Center for Biotechnology Information’s (NCBI) Basic Local Alignment Search Tool (BLASTx and BLASTn).

## Results and discussion

### DDRT-PCR of *M. sagu* tissues

**Fig. 2:**
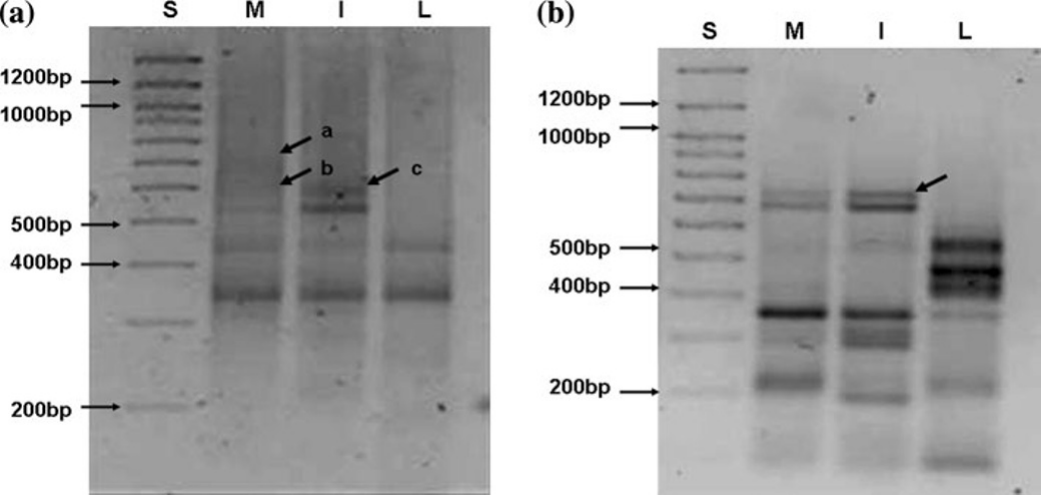
A 2.5% (w/v) agarose gel-electrophoresis of fragments amplified using (**a**) combination of oligo(dt)_15_ACP/AP2 primers. The fragments labelled as *a*, *b* and *c* represent fragments msAP21, msAP22 and msAP23, respectively. **b** Combination of oligo(dt)_15_ACP/AP3 primers. The *arrow* represents fragment msAP33. The *M*, *I* and *L* represent meristem, inflorescence and leaf cDNA samples, respectively. *S* represents a 100 bp Forever Ladder (Seegene)

The DDRT-PCR that was performed using oligo(dt)_15_ACP/AP2 primers combination generated several amplicons (indicated by arrows in Fig. 2a). Several amplicons were selected (a, b and c in Fig. 2a), cloned into a cloning vector and sequenced. From the BLAST analysis, the amplicons were determined to be derived from *chitinase* (msAP21, msAP22, and msAP23).

The DDRT-PCR method using oligo(dt)_15_ACP/AP3 primer combination generated several amplicons from which a few were selected, sequenced and one was identified to have high similarity to *chitinase* (msAP33) (indicated by arrow in Fig. 2b). The results showed *chitinase* is expressed in meristem and inflorescence tissues with higher expression in the inflorescence compared to meristem. However, no *chitinase* expression was detected in the leaves of *M. sagu*. These results were also in accordance with several researches in which they showed that *chitinase* was highly expressed in healthy floral organs and developing flowers, and either not expressed or at an extremely low level in vegetative organs (Neale et al. 1990; Wemmer et al. 1994; Hamel and Bellemare 1995; Harikrishna et al. 1996; Takakura et al. 2000).

### Isolation and analysis of *chitinase* cDNA from *M. sagu*

**Fig. 3:**
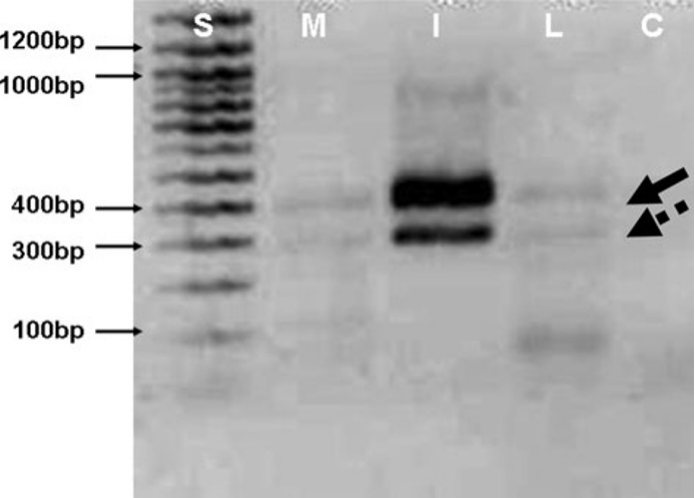
A 2.0% (x/v) agarose gel-electrophoresis of amplified 3′-end region of *chitinase* in *M. sagu* meristem, inflorescence and leaf tissues. *LaneS* represents a 100 bp Forever Ladder (Seegene). *LanesM*, *I* and *L* represent amplified product from meristem, inflorescence and leaf tissue, respectively. *Lane C* represents the negative control. *Arrow* indicates the 400 bp fragment and *dashed arrow* shows the 300 bp fragment

Several steps of RACE were undertaken to isolate *chitinase* cDNA from *M. sagu* tissues. The primers; emChi-f, Chi-sp1 and Chi-sp2, were designed for *chitinase* cDNA 3′RACE and 5′RACE. The 3′RACE of the meristem, inflorescence and leaf cDNA samples generated two bands of approximately 400 and 300 bp in all tissues (Fig. 3). Subsequent 5′RACE of the cDNA managed to produce an amplicon of approximately 900 bp (not shown). Amplification of 5′-end region was carried out only in the cDNA derived from inflorescence tissue of *M. sagu* because *chitinase* had been found to be expressed in higher levels in inflorescence tissue when compared to meristem and leaf tissues (Fig. 2).

**Fig. 4:**
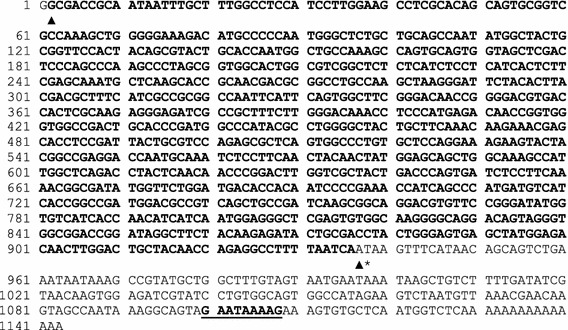
Nucleotide sequence of *chitinase* gene in *M. sagu* showing the open reading frame (ORF), stop codon dan polyadenylation site. *Asterisk* represents the stop codon and the polyadenylation signal is presented as *bold underlined* nucleotides. The ORF is located between the *arrowheads*

The 3′-end and 5′-end fragments of *chitinase* cDNA of *M. sagu* were combined and analysed using NCBI’s BLASTn software. Sequence analysis showed that amplified *chitinase* cDNA sequence of *M. sagu* (1,143 bp) share between 70 and 74% identity with *chitinase* mRNA of *Ananas comosus*, *Bambusa oldhamii*, *Bromus inermis*, *Citrus unshiu*, *Festuca arundinacea*, *Fragaria* x *ananassa*, *Hordeum vulgare*, *Medicago sativa*, *Leucaena leucocephala*, *Musa x paradisiaca*, *Nepenthes khasiana*, *Oryza sativa*, *Petroselinum crispum*, *Pinus halepensis*, *Secale cereale rscc*, *Triticum aestivum* and *Vitis vinifera* (Table 1). From the nucleotide size of 1,143 bp, the open reading frame (ORF) was determined to be 936 bp long with 312 deduced amino acids (Fig. 4). Comparison of the deduced amino acids of *M. saguchitinase* revealed that the ORF sequences exhibit similarity to *chitinase* domain family 19 and is closely related to class I *chitinase*.

**Table 1:** Comparison of nucleotide sequence similarity between *chitinase* cDNA of *M. sagu* with other plant species

Plant	Length (bp)	GenBank accession number	Homology (%)^a^
*Ananas comosus*	1,176	AB290909.1	74 (546/729)
*Bambusa oldhamii*	1,232	AY453406.1	73 (513/702)
*Bromus inermis*	1,168	AB428423.1	72 (654/900)
*Citrus unshiu*	1,101	AB364644.1	70 (617/878)
*Festuca arundinacea*	1,170	EU837265.1	74 (671/898)
*Fragaria* x *ananassa*	841	AF420225.1	71 (502/701)
*Hordeum vulgare*	998	M62904.1	73 (512/695)
*Medicago sativa*	1,267	U83592.1	70 (467/662)
*Leucaena leucocephala*	1,080	AF513017.2	73 (620/843)
*Musa x paradisiaca*	1,082	AY997529.2	71 (625/874)
*Nepenthes khasiana*	957	AY618886.1	73 (515/697)
*Oryza sativa*	1,208	EF122477.1	74 (648/873)
*Petroselinum crispum*	971	AF141372.1	71 (496/693)
*Pinus halepensis*	1,332	AY705804.1	70 (487/692)
*Secale cereale rscc*	1,018	AB051579.1	73 (518/705)
*Triticum aestivum*	1,148	AB029936.1	73 (659/901)
*Vitis vinifera*	945	DQ406689.1	73 (508/687)

^a^The percentages are based on BLASTn searches of the GenBank database. The numbers in brackets are the number of bases (query/subject) that have been compared

**Fig. 5:**
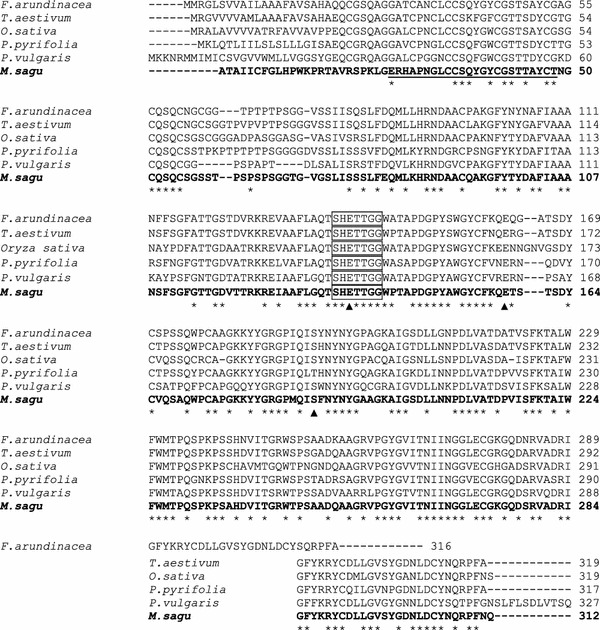
Multiple sequence alignment of the deduced amino acids of *MsChi*1 and class I *chitinase* from *Festuca arundinacea*, *Oryza sativa*, *Phaseolusvulgaris*, *Pyruspyrifolia* and *Triticumaestivum*. Identical amino acid residues are indicated by *asterisks*, the catalytic residues are denoted as *closed triangles*, amino acid for chitin binding domain are underlined and *chitinase* consensus sequence are indicated in*box*.*Dashed lines* are gaps introduced to maximize the identity

Sequence alignment of the deduced amino acid of *chitinase* from *M. sagu* (*MsChi*1) with class I *chitinase* from *Festuca arundinacea* (EU837265.1), *Oryza sativa* (Z29961.1), *Phaseolus vulgaris* (AY357300.2), *Pyrus pyrifolia* (FJ589783.1) and *Triticum aestivum* (AY437443.1) is shown in Fig. 5. A highly conserved amino acid region (SHETTGG), characteristic of *chitinase*, was also identified in *MsChi*1 of *M. sagu* (Fig. 5) therefore strengthening the cDNA to be of *chitinase* origin. In silico analysis of the amino acid sequence also indicated the presence of conserved domains. A glycoside hydrolase family 19 chitinase domain, that is involved in the hydrolysis of beta-1,4-*N*-acetyl-d-glucosamine linkages in chitin, was predicted to be present at amino acid 75–304. A chitin binding domain that is involved in the recognition and binding to chitin was detected from amino acid 24–47. Meanwhile, catalytic residues (amino acids 136, 158 and 188) and putative sugar binding sites (amino acids 136, 158, 186, 191–192, 267 and 279) for *MsChi*1 were also detected and are indicated in Fig. 5 (Marchler-Bauer et al. 2011).
